# The Two-Step Treatment for Giant Hepatic Hemangiomas

**DOI:** 10.3390/jcm10194381

**Published:** 2021-09-25

**Authors:** Angelo Della Corte, Rebecca Marino, Francesca Ratti, Diego Palumbo, Giorgia Guazzarotti, Simone Gusmini, Luigi Augello, Federica Cipriani, Guido Fiorentini, Massimo Venturini, Luca Aldrighetti, Francesco De Cobelli

**Affiliations:** 1 Department of Radiology, IRCCS San Raffaele Hospital, 20132 Milan, Italy; palumbo.diego@hsr.it (D.P.); guazzarotti.giorgia@hsr.it (G.G.); gusmini.simone@hsr.it (S.G.); augello.luigi@hsr.it (L.A.); decobelli.francesco@hsr.it (F.D.C.); 2 Experimental Imaging Center, IRCCS San Raffaele Scientific Institute, 20132 Milan, Italy; 3 Faculty of Medicine, University Vita-Salute San Raffaele, 20132 Milan, Italy; aldrighetti.luca@hsr.it; 4 Hepatobiliary Surgery Division, IRCCS San Raffaele Hospital, 20132 Milan, Italy; marino.rebecca@hsr.it (R.M.); ratti.francesca@hsr.it (F.R.); cipriani.federica@hsr.it (F.C.); fiorentini.guido@hsr.it (G.F.); 5 PhD School in Experimental Medicine, University of Pavia, 27100 Pavia, Italy; 6 Diagnostic and Interventional Radiology Department, Circolo Hospital, ASST Sette Laghi, 21100 Varese, Italy; massimo.venturini@uninsubria.it

**Keywords:** giant hepatic hemangioma, laparoscopic surgery, radiologic arterial embolization

## Abstract

The aim of the present study is to analyze the feasibility and the impact of a two-step approach in the treatment of giant hemangiomas (GH) i.e., exceeding 10 cm in maximum diameter, consisting of transarterial embolization (TAE) followed by laparoscopic liver resection (LLR). Ten patients with 11 GH were treated with TAE and subsequent LLR between 2017 and 2020 (Group A). A matched cohort of 10 patients with GH treated with upfront LLR between 2014 and 2017 was identified for comparison (Group B). Data were analyzed regarding intraoperative and postoperative outcomes, including successful completion of LLR, morbidity, and mortality. Successful microparticle embolization of the GH-feeding arteries was performed in all patients in group A. In three cases a liquid embolic agent (Squid-18) was also injected to obtain complete embolization. No complications were observed after TAE. Successful surgery was performed after a mean time interval of 2.2 days from TAE without any case of conversion to laparotomy. Statistically significant differences between group A and group B were found in intraoperative blood loss (250 ± 200 vs. 400 ± 300 mL, *p* = 0.039), operative time (245 ± 60 vs. 420 ± 60 min, *p* = 0.027), and length of stay (5 ± 1 vs. 8 ± 2 days, *p* = 0.046). Our data suggest that two-step TAE + LLR might be a safe and effective option for surgical treatment of GH >10 cm.

## 1. Introduction

Hepatic hemangiomas are the most common vascular lesions of the liver, with a prevalence varying from 0.4% to 20% in the general population [1]. Generally, small (<5 cm in largest diameter) asymptomatic hemangiomas are referred to surveillance imaging. Three scenarios, though, require prompt intervention: (i) rapid tumor growth causing disabling symptoms, (ii) complications, such as *Kasabach–Merrit* syndrome or sudden rupture, and (iii) diagnostic uncertainty [2]. Management of patients with giant hemangiomas (GH) exceeding 10 cm in diameter is currently controversial. Despite prophylactic surgical treatment, historically advocated as the *standard of care* to avoid potentially life-threatening complications [3], the prevention of rupture alone is not considered a straightforward indication for surgical removal of an asymptomatic lesion [4], also taking into account potential postoperative complications.

In recent years, the wider adoption of the laparoscopic approach has led to dramatic improvements in surgical outcomes, with lower stress response, reduced blood loss and morbidity, earlier recovery, and improved cosmetic outcomes when compared to open liver resections as its main advantages [5].

Some case series of successful laparoscopic liver resections (LLRs) for selected hepatic hemangiomas are currently available in the literature [6,7,8,9,10,11,12] yet all authors highlight several technical difficulties; high rates of conversion to open surgery and massive intraoperative blood loss are the most challenging. In addition, there is growing evidence that size itself is among the most important risk factors associated with uncontrollable bleeding and limited intrabdominal surgical workspace [2,13,14], making LLR a particularly challenging choice for the treatment of GHs exceeding 10 cm.

In order to address this major issue, the limited inhomogeneous manuscripts available are single case reports that propose a multidisciplinary approach consisting of preoperative transarterial embolization (TAE) followed by LLR [15,16].

The biological rationale of such an approach is that hemangiomas microscopically consist of a vascular bed, coated by a single layer of flat endothelium; preoperative embolization directly affects this vascular bed by reducing blood inflow, thus, significantly lowering the risk of severe hemorrhage during LLR [16]. 

The aim of the present study is to systematically analyze the clinical efficacy and safety of a two-step approach for the treatment of GHs exceeding 10 cm, consisting of preoperative TAE followed by LLR, and to evaluate its outcomes in comparison to upfront stand-alone LLR.

## 2. Material and Methods

### 2.1. Study Design

This study was carried out in accordance with the Declaration of Helsinki of the World Medical Association and approved ethically by the Ethics Committee of the San Raffaele Hospital, Milan (Italy). Written informed consent was obtained from all patients.

A retrospective analysis was performed on a prospectively collected database including consecutive patients with giant hepatic hemangiomas (>10 cm in maximum diameter) treated with transarterial embolization (TAE) at the Department of Interventional Radiology, planned for laparoscopic resection at the Division of Hepatobiliary Surgery between November 2017 and October 2020 (Group A).

After diagnosis, the indication for combined treatment was shared in a multidisciplinary meeting including at least one hepatobiliary surgeon and one interventional radiologist. All patients underwent preoperative computed tomography (CT) scan to identify the main feeding arteries, eventual additional feeders and to evaluate the presence of anatomical variants. Patients were included if: (1) they were >18 years old; (2) they had a diagnosis of giant hepatic hemangioma (>10 cm) confirmed by ultrasound, CT, or Magnetic Resonance Imaging (MRI); (3) preoperative CT scan images were available; 4) they underwent TAE prior to laparoscopic resection. Exclusion criteria were: (1) uncorrectable coagulopathy (platelet count < 50,000/L and/or international normalized ratio > 1.5); (2) pregnancy; (3) stage > 3 chronic kidney disease; (4) unavailability of pre-procedural imaging. 

Using a prospectively maintained institutional database, a matched cohort of patients with GH treated with upfront surgical resection between January 2014 and October 2017 was retrieved (Group B). Inclusion criteria for the comparison cohort were: (1) age > 18 years; (2) imaging diagnosis of giant hepatic hemangioma (>10 cm); (3) scheduled to receive laparoscopic approach.

The primary endpoint of this study was to evaluate the safety and feasibility of preoperative TAE. The secondary endpoint was to compare the surgical outcomes in group A (TAE + LLR) versus group B (stand-alone LLR).

### 2.2. Transcatheter Arterial Embolization

The day before TAE, all available patient imaging was reviewed to ensure accurate planning of the procedure. In all cases, TAE was performed by experienced interventional radiologists. The procedure and the advantages and disadvantages of TAE were explained to the patient before obtaining informed consent.

**Figure 1:**
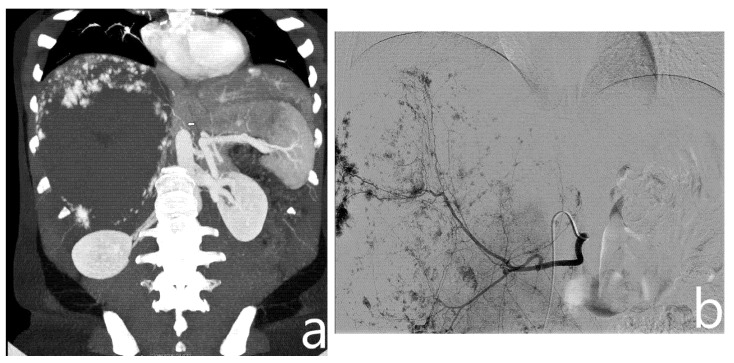

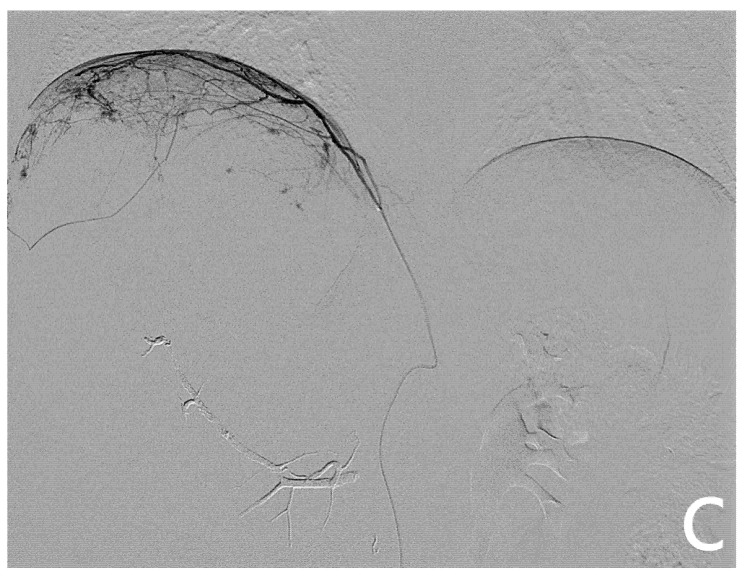
On preoperative CT (**a**), a giant hemangioma of the right liver lobe is identified, with evidence of vascular afference from the right phrenic artery (arrow). On angiography, after identification and embolization of the right hepatic artery (**b**), right phrenic artery is selectively catheterized and embolized with microparticles (**c**).

With the patient in the supine position, right femoral access was obtained using a 4F arterial sheath (Terumo Corporation, Tokyo, Japan) with the Seldinger technique. Based on a preoperative CT scan, a 4F catheter (Cordis, Santa Clara, CA, USA), via the arterial sheath, was used to obtain selective angiography of the main arteries involved in the vascularization of the hemangioma (mainly celiac trunk, superior mesenteric artery, and phrenic arteries, Figure 1); if necessary, an aortography was performed using a 4F Pig catheter (Cordis, Santa Clara, CA, USA).

**Figure 2:**
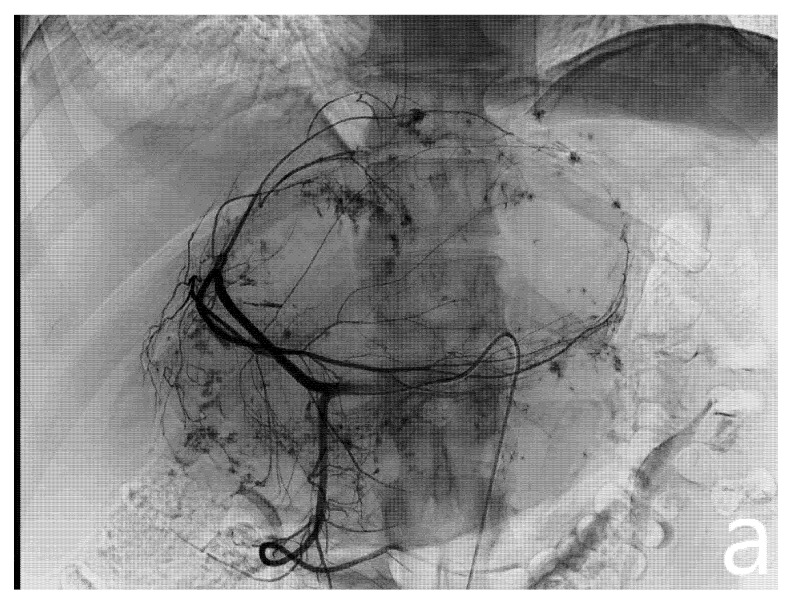

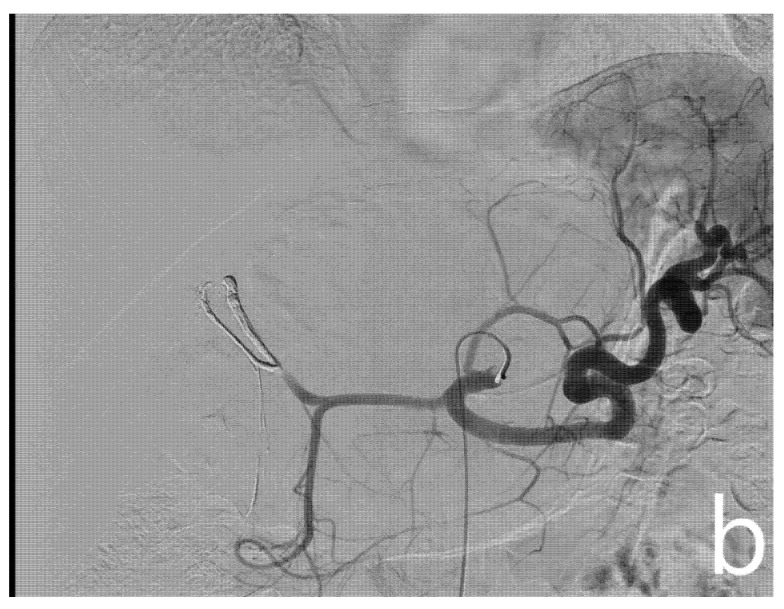
Pre- and post-embolization angiography. A giant hemangioma with vascular afference from the left hepatic artery originating independently from the celiac trunk is identified (**a**). Embolization is performed with microparticles and Squid 18 to achieve complete lesion devascularization (**b**).

After confirmation of the feeding arteries, superselective catheterization of the single feeding vessel was obtained using a microcatheter (Boston Scientifics, Marlborough, MA, USA); embolization was achieved using polyvinyl alcohol particles (PVA), whose size varied, depending on the caliber of the feeding artery. In patients planned for major hepatectomies, whenever a wide feeding artery was identified, ethylene-vinyl alcohol copolymer (EVOH)-based liquid embolic agent (Squid Peri, Emboflu, Switzerland) was used to complete the embolization (Figure 2).

Post-procedure, vital signs, oxygen saturation, routine blood investigations, liver and kidney function were monitored. Particular attention was paid to the lower limb pulses, temperature, and color of the skin. All periprocedural complications, classified according to the Cardiovascular and Interventional Radiological Society of Europe (CIRSE) Classification System [17], were systematically registered.

### 2.3. Surgical Technique

With the patient placed in a modified French position (both inferior and superior limbs abducted, with the operative table turned left and in reverse Trendelenburg position), a standardized setup of five 10 mm trocars was adopted. No trans-thoracic ports were used to avoid increased morbidity (pneumothorax, pleural effusion, and postoperative pain).

After an intraoperative ultrasound, liver transection was conducted with a combined use of energy devices, ultrasonic dissector, and bipolar forceps. An extracorporeal Pringle maneuver was routinely used to control intraoperative bleeding. The surgical specimen was extracted in a retrieval bag through a Pfannenstiel incision.

### 2.4. Perioperative Management

The ERAS fast-track protocol was implemented into clinical practice to enhance functional recovery. Characteristics of the institutional protocol are described elsewhere [18]. The patient is considered functionally recovered when all of the following criteria are fulfilled: adequate pain control with oral analgesics, independent mobilization (mobile at preoperative level), tolerance of liquids and solid food, normal or decreasing serum bilirubin, no intravenous fluids, absence or successful treatment and recovery from any complications.

### 2.5. Outcome Evaluation

Data regarding the general characteristics of patients and disease were recorded. Different types of hepatic resection were classified according to the Brisbane Classification [19]. Intraoperative and postoperative outcomes were evaluated including tumor rupture, morbidity, and mortality. Postoperative complications were reviewed for 90 days following liver resection and were graded retrospectively according to the Clavien–Dindo classification of surgical complications [20]. Postoperative mortality was defined as any death within 90 days after resection. 

Technical success was defined as an effective laparoscopic resection of the GH.

### 2.6. Statistical Analysis

Categorical variables were expressed as frequencies and percentages; continuous variables were expressed as mean plus the standard deviation. All analyses were performed using the statistical package SPSS v25.0 (IBM, Armonk, NY, USA). The distribution of categorical variables between the two study groups was assessed through Chi-square analysis; continuous variables were compared through the Mann–Whitney U-test. The significance level for all parameters was set at *p* ≤ 0.05.

## 3. Results

### 3.1. Baseline

Group A (TAE + LLR) consisted of ten patients, including a total of eleven giant hepatic hemangiomas; Group B (stand-alone LLR) consisted of ten patients with ten giant hemangiomas.

The mean age of presentation in group A was 45 years while the mean BMI was 23.2. Just one case out of the ten cases analyzed occurred in a male patient resulting in a primarily female-dominated group (90% vs. 10%). The majority of the patients (60%) were free from underlying liver disease or other comorbidities. The most common underlying liver condition was steatosis (*n* = 2; 20%). Previous abdominal surgery was reported in two cases (20%). Regarding tumor features, the mean greatest diameter was 13.8 cm. Eight lesions were localized in the right hepatic lobe (72.7%), while the remaining three lesions were localized in the left hepatic lobe (27.3%). All the lesions were localized less than 1 cm away from intrahepatic vessels >3 mm. Four GHs (36.4%) involved at least three liver segments.

Analysis of these baseline characteristics did not highlight any significant differences between groups A and B (Table 1).

### 3.2. Embolization Outcome

Embolization outcomes are summarized in Table 2. Three lesions in the study cohort showed evidence of arterial supply from the right phrenic artery, combined with either the right hepatic artery (*n* = 2, 18.2%) or the left hepatic artery (*n* = 1, 9.1%).

All the target vessels successfully underwent microparticle embolization. In three cases (30%), Squid 18 was used to complete embolization of the right (*n* = 1) or left hepatic artery (*n* = 2). All the procedures were well-tolerated with no complications.

### 3.3. Surgical Outcomes

The intra- and post-operative outcomes of the procedures in the two groups are summarized in Table 3.

After a mean interval of two days from embolization, all patients in group A underwent successful laparoscopic resection without the need for conversion to open surgery (technical success: 100%). Five patients (50%) required a minor resection (left lateral sectionectomy, right posterior sectionectomy, and single-segment anatomic liver resection) while the other 50% underwent major resections (right and left hepatectomies). In all cases, a review of the surgical specimen confirmed the diagnosis of hemangioma and demonstrated tumor necrosis.

A Pringle maneuver was performed in all cases. Mean operative time and blood loss were 145 min and 250 mL, respectively, while the mean length of hospital stay was five days. No intraoperative adverse events were recorded. In particular, no cases of tumor rupture occurred (0%). No major complications (Clavien–Dindo ≥ III) were reported; two patients (20%) experienced a decrease in Hb levels requiring intra- and post-operative whole blood transfusions (grade II).

In group B, two patients experienced significant intraoperative bleeding leading to conversion to laparotomy (20%). Three patients required intraoperative blood transfusions, and four postoperative blood transfusions (Clavien-Dindo grade II). One patient was diagnosed with biliary leakage treated with a percutaneous biliary drainage placement (Clavien–Dindo grade III). Statistically significant differences between group A and group B were found in intraoperative blood loss (250 ± 200 vs. 400 ± 300 mL, *p* = 0.039), operative time (245 ± 60 vs. 420 ± 60 min, *p* = 0.027), and length of stay (5 ± 1 vs. 8 ± 2 days, *p* = 0.046).

## 4. Discussion

In all cases, TAE was well-tolerated with no procedure-associated complications. In order to further decrease the risk of intraoperative bleeding that has been notoriously associated with GH surgery [21,22,23], preoperative TAE was associated with intraoperative routine Pringle maneuver. Mean operative time (145 min), blood loss (250 mL), and length of hospital stay (five days) were acceptable and consistent with LLR advantages. No conversion to open surgery and no intraoperative adverse events were described; moreover, a significant reduction in intraoperative blood loss, operative time, and length of stay were observed with respect to patients treated with upfront LLR.

In recent years, an unparalleled growth of laparoscopic procedures in the field of liver resections has been witnessed [24,25]. The well-established advantages of a pure laparoscopic approach such as decreased postoperative pain, shorter length of hospital stay, better preservation of abdominal wall integrity, and earlier return to daily activities are readily perceived when applied to benign hepatic lesions [5]. 

As reflected in our study cohort, the majority of patients affected by giant hemangiomas are young females with healthy livers and no history of previous abdominal surgery [2]. If on one hand these patients’ characteristics are usually linked to reduced comorbidities’ rate, on the other hand, the large tumor volume and its vascularization are associated with increased surgical complexity [22,23]. 

The literature regarding laparoscopic liver resection (LLR) applied to GH cases is extremely scarce and no consensus is established on whether GH should be considered a standard indication for performing laparoscopic surgery. 

The limited case series of successful LLRs of GHs exceeding 5 cm available [6,11,12] confirmed several technical difficulties encountered in our matched comparison cohort, i.e., conversion to open surgery and massive intraoperative bleeding.

In particular, Jinhuan et al. [11] retrospectively analyzed data from 58 patients affected by GH > 10 cm, of which 28 were categorized as a “High-difficulty group” based on proximity to large vessels, lesion diameter, segments involvement, and with characteristics comparable to our study cohort. Despite routine intraoperative vascular occlusion, higher intraoperative bleeding, and length of hospital stay were observed in the high-difficulty group, justifying the need for better control of bleeding in selected cases.

Ardito F et al. reported no increased numbers of LLR for benign liver disease despite an evident overall growth of LLR in Italy [26]. Following its first literature report by Yamamoto et al. [15], the role of TAE has been investigated as a stand-alone treatment of GHs, with reported satisfying results in terms of GH size reduction and symptom control [27]. 

Nevertheless, TAE outcomes are still controversial due to the increased risks of ischemia, infections, intracavitary bleeding, ectopic embolization, and biliary damage [28,29]. In addition, in cases of highly vascularized lesions, well-structured collateral circulation and vascular recanalization are common. These factors might lead to recurrence and nullify procedure effectiveness [2].

Pre-operative TAE prior to LLR of GHs has only been described in a few case reports [7,8,9]. However, given the anecdotal nature of these studies, no consistent information is provided regarding the embolizing materials, limiting the reproducibility of these data. In particular, Veerankutty et al. [9] did not report a substantial benefit of embolization concluding that this was due to the presence of collateral vascularization. A single study in the literature compared the outcome of open liver resection of GHs with or without preoperative embolization, showing no significant differences between the two groups [16].

In our study, the diagnostic interventional approach followed a standardized protocol, from feeding arteries identification on multi-modal imaging to the choice of embolizing materials. In this regard, PVA particles ranging from 355 to 500 μm were chosen in order to achieve distal vascular occlusion, minimizing non-target ischemic effects, and the development of collateral vascularization. Liquid embolic agents were used to achieve more proximal embolization in selected patients planned for major hepatectomies, whenever a satisfactory flow reduction was not achieved with microparticles alone. In particular, although the use of N-butyl cyanoacrylate would be another good option, the use of Squid 18, already described in the abdominal area [30,31,32], was based on the operators’ preference since it allows the formation of a stable cast within wide GH-feeding arteries whilst minimizing risks of non-target embolization or catheter retention in a setting of relatively reduced flow.

Whenever identified, selective embolization of two different sources was performed. The effective devascularization not only lowered the risk of traumatic rupture of the mass during laparoscopic mobilization but also optimized intrabdominal surgical workspace by increasing the freedom of motion during surgery. In addition, selective embolization of phrenic branches guaranteed a significant reduction in diaphragmatic vascular afferents that are often difficult to control during laparoscopy and may constitute a cause of bleeding during mobilization phases. In our cohort, preoperative TAE mitigated LLR technical difficulties when applied to GH whilst taking advantage of the well-known benefits of LLR. 

The potential limitations of our study are its retrospective nature and the limited number of cases. A prospective randomized trial comparing the use of preoperative TAE and subsequent surgery versus upfront surgery may be useful to provide further insights into the role of this synergistic association of interventional radiology and liver resection.

In conclusion, despite LLR having only been initially recommended for lesions < 5 cm [33], our results suggested that two-step TAE + LLR might be a safe and effective option for surgical treatment of GH > 10 cm.

## Figures and Tables

**Table 1:** Population baseline characteristics.

Population Baseline Characteristics (*n* = 10)	Group A (*n* = 10)	Group B (*n* = 10)	*p*
Age (years) *	45 ± 10	43.7 ± 8.5	0.67
BMI *	23.2 ± 4.5	24.1 ± 5.7	0.82
Female/Male	9 (90%)/1 (10%)	10 (100%)/0 (0%)	0.56
Maximum GH diameter (cm) *	13.8 ± 3	12.2 ± 1.5	0.77
GH located in the Right Hepatic Lobe	6 (60%)	4 (40%)	0.48
GH located in the Left Hepatic Lobe	3 (30%)	4 (40%)	0.54
Bilobar	1 (10%)	2 (20%)	0.58
Proximity to large vessels (<1 cm)	11 (100%)	10 (100%)	ns
≥3 Segments involved	4 (36%)	4 (40%)	0.62
Liver Steatosis	2 (20%)	1 10%)	0.58
Previous Abdominal Surgery	2 (20%)	1 (10%)	0.58

BMI = Body Mass Index; GH = Giant Hemangioma; ns = non-significant; * Values expressed as mean ± SD.

**Table 2:** Embolization Outcome.

Embolization Outcome	
Vascularization from RHA only	5 (45.4%)
Vascularization from LHA only	3 (27.3%)
Vascularization from RHA + RPA	2 (18.2%)
Vascularization from LHA + RPA	1 (9.1%)
Embolization performed with PVA (355–500 μm)	7 (70%)
Embolization performed with PVA (355–500 μm) + Squid-18	3 (30%)
Complications	0 (0%)
Time from embolization to surgery (days) *	2.2 ± 0.7

RHA = Right hepatic artery; LHA = Left hepatic artery; RPA = Right Phrenic Artery; PVA = Polyvinyl Alcohol; * Values expressed as mean ± SD.

**Table 3:** Surgical Outcome.

Surgical Outcome			
	Group A (*n* = 10)	Group B (*n* = 10)	*p*
Pringle Maneuver, *n* (%)	10 (100%)	10 (100%)	ns
Right hepatectomy §	2 (20%)	3 (30%)	0.61
Left hepatectomy §	1 (10%)	3 (30%)	0.29
Left lateral sectionectomy §	1 (10%)	1 (10%)	ns
Right posterior sectionectomy §	3 (30%)	1 (10%)	0.29
Single segment anatomic resection §	2 (20%)	2 (20%)	ns
Associated procedures, *n* (%)	0 (0%)	2 (20%)	0.24
Intraoperative adverse events, *n* (%)	2 (20%)	2 (20%)	ns
Intraoperative blood transfusions, *n* (%)	2 (20%)	3 (30%)	0.61
Postoperative blood transfusions, *n* (%)	2 (20%)	4 (40%)	0.31
Conversion, *n* (%)	0 (0%)	2 (20%)	0.24
Morbidity, *n* (%)	2 (20%)	5 (50%)	0.18
Complications *n* (%)	Grade I 0 (0%)	Grade I 0 (0%)	ns
	Grade II (20%)	Grade II 4 (40%)	0.31
	Grade ≥ III (0%)	Grade ≥ III 1 (10%)	0.32
Operative time (min) *	145 ± 60	420 ± 60	0.027
Intraoperative Blood Loss (mL) *	250 ± 200	400 ± 300	0.039
Length of Stay (days) *	5 ± 1	8 ± 2	0.046
Mortality, *n* (%)	0 (0%)	0 (0%)	ns

* Values expressed as mean ± SD; § Definition according to Brisbane classification; ns = non-significant.

## Data Availability

The data presented in this study are available on request from the corresponding author. The data are not publicly available since they are covered by institutional privacy policies.
